# Hereditary angioedema: health-related quality of life in Canadian patients as measured by the SF-36

**DOI:** 10.1186/s13223-016-0176-3

**Published:** 2017-01-19

**Authors:** Nina Lakhani Jindal, Elaine Harniman, Nieves Prior, Elia Perez-Fernandez, Teresa Caballero, Stephen Betschel

**Affiliations:** 1 Division of Clinical Immunology and Allergy, St. Michael’s Hospital, Bond Street, 4 CC Specialty Clinics, Toronto, ON M5B 1W8 Canada; 2 Musculoskeletal Health and Outcomes Research, Li Ka Shing Knowledge Institute, St Michael’s Hospital, Toronto, ON Canada; 3 Allergy Department, Hospital Universitario Severo Ochoa, Leganés, Madrid Spain; 4 Research Unit, Hospital Universitario Fundación Alcorcón, Madrid, Spain; 5 Allergy Department, Hospital La Paz Institute for Health Research (IdiPaz), Madrid, Spain; 6 Biomedical Research Network on Rare Diseases (CIBERER, U754), Madrid, Spain

**Keywords:** Hereditary angioedema, HAE, Quality of Life, SF-36, C1-inhibitor

## Abstract

**Background:**

Hereditary angioedema (HAE) is a rare but serious condition characterized by recurrent spontaneous attacks of angioedema affecting superficial tissues of upper respiratory and gastrointestinal tracts. The potentially fatal and disfiguring nature of HAE impacts the health-related quality of life (HRQoL) of patients with this condition.

**Objectives:**

To assess the health-related quality of life of Canadian patients with HAE using the 36-item Short-Form Health Survey (SF-36v2).

**Methods:**

Twenty-one patients living in Canada over age 18 with known diagnosis of hereditary angioedema due to C1-INH deficiency (HAE), completed the SF-36v2 (generic HRQoL questionnaire). Results were compared to Canadian normative data by converting the SF-36 scores into z scores.

**Results:**

The SF-36v2 showed a significant reduction in general health (p = 0.0063) in patients with HAE when compared with healthy Canadians. Percentage of patients with z scores below 0.8 (large effect) was 47.6% for general health subscale, 33.3% for bodily pain and vitality subscales and 28.6% for physical component scores. Mean scores of eight dimensions ranged from 57.7 to 88.9. Mean Physical and mental component scores were 49.1 and 50.4. Internal consistency of evaluation was demonstrated by Cronbach’s alpha value above 0.7 for all scales. General perception of health was significantly different in these patients, compared to Canadian normative data.

**Conclusions:**

This study of Canadian patients with HAE shows that General Health is most frequently affected followed by Bodily Pain and Vitality, as measured by SF-36v2. The SF-36v2 offers valuable insight to assess quality of life in patients with HAE, however a larger number of Canadian patients and specific tools for assessment are needed for better evaluation.

## Background

Hereditary angioedema (HAE) is a rare but serious condition due to a deficiency in serine protease inhibitor C1 inhibitor (C1INH), leading to accumulation of bradykinin [1]. It is characterized by recurrent attacks of swelling affecting any part of the body, most commonly involving the extremities, face, genitals, gastrointestinal tract and upper airways. HAE is an autosomal dominant condition caused by mutations in the SERPING1 gene, with 25% of cases representing a de novo mutation, without a family history of disease [1, 2]. The estimated prevalence of HAE is between 1:30,000 and 1:80,000, with no predilection for gender, race or ethnicity [1].

Episodes of swelling follow a stereotypic pattern in which worsening of bradykinin-mediated edema occurs over 24 h, peaks and slowly resolves over the subsequent 48–72 h [1, 3]. Certain trigger factors have been identified including emotional stress, local trauma, dental or medical procedures, infection, menstruation and the use of hormonal contraception [1, 2].

Despite this, severity of attacks remains unpredictable at onset and if affecting the upper airways, can lead to obstruction, asphyxiation and death [4].

Gastrointestinal attacks can cause significant pain and often are mistaken for acute abdomen, leading to unnecessary surgical intervention [5]. Edema affecting the skin and extremities causes disfigurement and if severe, compartment syndrome [1].

The unpredictable, potentially fatal as well as disfiguring nature of the disease impacts the health-related quality of life (HRQoL) of patients with this condition, however few publications have described the quality of life in patients with HAE. The Short Form 36 (SF-36v2) is a standardized patient reported survey of patient health, and is a measure of health status commonly used in health economics.

The aim of this study was to assess the health-related quality of life of Canadian patients with hereditary angioedema using the 36-item Short-Form Health Survey (SF-36v2).

## Methods

### Ethical aspects

Study protocol was approved by the Research and Ethics Board of St. Michael’s Hospital, Toronto and the Faculty of Medicine at the University of Toronto. Patients were recruited through the Canadian HAE patient network and treating physicians across Canada. Informed consent was obtained from all patients before study questionnaires were completed.

### Patient population

Twenty-one patients living in Canada over the age of 18 with a known laboratory confirmed diagnosis of hereditary angioedema due to C1-INH deficiency (HAE) were included in the study (Table 1).

**Table 1:** Descriptive analysis and Cronbach’s alpha values for the eight subscales and two summary scales of the SF-36 in 21 hereditary angioedema patients

Domain	Minimum	Maximum	Median	Mean	Standard deviation	Cronbach’s alpha	Floor n (%)	Ceiling n (%)
Physical functioning (PF)	40.00	100.00	95.00	87.86	16.25	0.86	0 (0)	8 (38.10)
Role physical (RP)	0.00	100.00	93.75	84.23	23.19	0.91	1 (4.76)	7 (33.33)
Bodily pain (BP)	22.00	100.00	74.00	67.90	21.79	0.89	0 (0)	3 (14.29)
General health (GH)	5.00	100.00	62.00	61.05	23.39	0.84	0 (0)	1 (4.76)
Vitality (VT)	25.00	81.25	62.50	57.74	18.32	0.83	0 (0)	0 (0)
Social functioning (SF)	0.00	100.00	100.00	85.12	27.28	0.95	1 (4.76)	13 (61.90)
Role emotional (RE)	16.67	100.00	100.00	88.89	24.49	0.94	0 (0)	16 (76.19)
Mental health (MH)	25.00	90.00	80.00	76.19	16.95	0.89	0 (0)	0 (0)
Physical summary (PCS)	34.17	63.21	48.62	49.13		N/A	N/A	N/A
Mental summary (MCS)	15.99	65.88	54.60	50.35		N/A	N/A	N/A

## Data collection

### Patients completed the generic SF-36v2

SF-36v2 is a generic health questionnaire measuring eight health domains including physical functioning (PF), limitations in daily role functioning due to physical problems (RP), bodily pain (BP), general health (GH), vitality (VT), social functioning (SF), limitations in daily role functioning due to emotional problems (RE), mental health (MH) as well as an item asking respondents about health change over the past year. Scores for each domain can range from 0 to 100, higher scores indicating better health state. Scores on each scale were calculated based on the ‘half item rule’. Two summary scores were also calculated from the eight domains: the physical component summary (PCS) and the mental health component summary (MCS). Summary scores are normalized to age-matched controls and scored to have a mean of 50 and standard deviation of 10.

### Statistical analysis

Descriptive statistics for demographic information were calculated. Results were compared to Canadian normative data by converting the SF-36v2 scores into z-scores. Z-scores with values above 0.80 are considered to have large effects.

Each person’s score was standardized by subtracting the mean value and dividing by the standard deviation for the Canadian norms of the same age and gender group [6]. This z-score has a mean of 0 and a standard deviation of 1. This method of examining differences between samples is similar to calculating effect sizes [7]. Thus, the z-scores can be interpreted in the same way with values above 0.80 considered large effects. We used a one-sample *t* test, with zero as test value, to determine if significant differences (p < 0.05) from age- and gender-matched Canadian norms existed.

Cronbach’s alpha was used to assess the internal consistency of each subscale and a value equal to or greater than 0.7 was considered acceptable. Data were analyzed using SAS software, version 9.4 (SAS Institute, Cary, NC).

## Results

Twenty-one patients were included in the study. The majority of patients were female (20 = 95.2%), from an urban area (15 = 71.4%), and had an education level above high school (15 = 71.4%) and a medium–low socio-economic level (8 = 44.4%). The mean age was 42.3 years (SD 13.7 years).

The percent of patients with a z score below 0.8 (large effect) was 47.6% for General Health subscale, 33.3% for Bodily Pain and Vitality subscales, and 28.6% for the physical component summary score. The SF-36v2 showed there was a significant reduction in general health (p = 0.0063) in patients with hereditary angioedema (HAE) when compared with healthy Canadians (Table 2). The mean scores of the eight dimensions ranged from 57.7 to 88.9. The mean Physical and Mental Component Summary Scores were 49.1 and 50.3. General perception of health was significantly different in this patient population, compared to Canadian normative data. There were no significant relationships between SF-36v2 scores and age or education level.

**Table 2:** SF36v2 HAE patients compared to Canadian normative data [6]

SF-36 domain	≥0.8 SD below	0.5–0.8 SD below	within 0.5 SD of mean	0.5–0.8 SD above	≥0.8 SD above	Mean (sd)	p value^a^
Physical functioning	14.3	14.3	23.8	42.9	4.8	0.06 (0.73)	0.6926
Role-physical	4.8	4.8	61.9	28.6	0.0	0.08 (0.71)	0.6197
Bodily pain	33.3	9.5	42.9	0.0	14.3	−0.29 (0.98)	0.1991
General health	47.6	4.8	33.3	4.8	9.5	−0.95 (1.42)	0.0063
Energy/vitality	33.3	9.5	23.8	28.6	4.8	−0.29 (1.05)	0.2207
Social functioning	14.3	4.8	19.1	42.9	19.1	0.02 (1.40)	0.9607
Role-emotional	14.3	0.0	9.5	76.2	0.0	0.20 (0.81)	0.2669
Mental health	14.3	4.8	33.3	19.1	28.6	0.02 (1.13)	0.9303
Physical component scale	28.6	14.3	33.3	9.5	14.3	−0.23 (0.99)	0.3027
Mental component scale	19.1	0.0	33.3	33.3	14.3	−0.05 (1.39)	0.8825

^a^From one sample *t* test

Cronbach’s alpha exceeded 0.70 for all scales, very high ceiling effects occurred for the SF-36 RE (76.2%) and SF subscales (61.9%) followed by the PF (38.1%) and RP (33.3%) subscales. Cronbach’s alpha for these four subscales ranged from 0.86 to 0.95 while the range for the other four subscales was 0.83–0.89. Two items (sf8 and sf3d) each had one missing value (4.8%).

## Discussion

Hereditary angioedema is a rare but serious condition, in which affected patients suffer a significant burden of disease, similar to that of severe asthma or Crohn’s disease [8]. Additionally, patients with HAE deal with the uncertainties and unpredictable nature of episodic attacks of angioedema.

Few studies have evaluated the quality of life in patients with HAE [3, 9]. In this study, Canadian patients exhibited an impaired quality of life, as measured by the standardized SF-36v2, particularly in the domains of general health, bodily pain and vitality. In previous data, patients with HAE reported decreased mental and physical health as well as social effects compared to a normal population, in keeping with our study of Canadian patients [3, 9, 10].

In both the study by Gomide [10] and our study, the SF-36 subscales with the lowest median values were General Health, Bodily Perception and Vitality (40, 52, and 60 respectively for the Gomide [10] study and 62.0, 74.0, and 62.5 respectively for our study). The median value for the SF subscale differed between the two studies (median value of 50 vs. 100). A more recent Danish study by Aabom et al. showed mean SF-36v2 scores corresponding with population norms, likely explained by differences in treatment options and access to around the clock counseling for Danish patients [11].

The high Cronbach’s alpha for all SF-36 scales indicates a high internal consistency. However, the high alpha coefficients observed might be partly due to the ceiling effects observed in some subscales (mainly RE and SF, but also PF and RP). These ceiling effects could be related to a low sensitivity of these subscales or to nearly no affection of these subscales in Canadian HAE patients.

Health-related quality of life questionnaires are developed to evaluate individual patient health status, assess the cost-effectiveness of an intervention or treatment, and to monitor disease burden. Several questionnaires have been developed to evaluate health related quality of life and help establish resource allocation [12]. The standardized SF-36v2 questionnaire can be a useful tool in the assessment of quality of life in patients with HAE, which allows comparison to other diseases, however a need exists for disease-specific surveys, to serve as a more precise indicator of disease burden. Disease-specific surveys can serve as a point of comparison with generic questionnaires and further evaluate particular aspects of disease severity. A specific HAE QOL questionnaire has been developed in Spain and translated and validated in other countries [13, 14].

## Conclusion

This study of Canadian patients with HAE shows that General Health is most frequently affected followed by Bodily Pain and Vitality, as measured by SF-36v2. The SF-36v2 offers valuable insight to assess quality of life in patients with HAE, however a larger number of Canadian patients and specific tools for assessment are needed for better evaluation.

## Abbreviations

HAE: hereditary angioedema
SF-36v2: short form health survey 36 version 2
HRQoL: health-related quality of life
C1INH: C1 inhibitor
PF: physical functioning
RP: limitations in daily role functioning due to physical problems
BP: bodily pain
GH: general health
VT: vitality
SF: social functioning
RE: limitations in daily role functioning due to emotional problems
MH: mental health
PCS: physical component summary
MCS: mental health component summary
